# Perspectives of policymakers and health providers on barriers and facilitators to skilled pregnancy care: findings from a qualitative study in rural Nigeria

**DOI:** 10.1186/s12884-020-03493-8

**Published:** 2021-01-06

**Authors:** Ogochukwu Udenigwe, Friday E. Okonofua, Lorretta F. C. Ntoimo, Wilson Imongan, Brian Igboin, Sanni Yaya

**Affiliations:** 1 School of International Development and Global Studies, University of Ottawa, Ottawa, Ontario Canada; 2 Women’s Health and Action Research Centre, KM 11 Lagos-Benin Expressway, Igue-Iyeha, Benin City, Edo State Nigeria; 3 Centre for Excellence in Reproductive Health Innovation, Benin City, Nigeria; 4 Federal University Oye-Ekiti, Oye-Ekiti, Nigeria; 5 The George Institute for Global Health, Imperial College London, London, UK

**Keywords:** Maternal health, Skilled pregnancy care, Rural, Global health, Nigeria, Barriers, Facilitators

## Abstract

**Background:**

The uptake of skilled pregnancy care in rural areas of Nigeria remains a challenge amid the various strategies aimed at improving access to skilled care. The low use of skilled health care during pregnancy, childbirth and postpartum indicates that Nigerian women are paying a heavy price as seen in the country’s very high maternal mortality rates. The perceptions of key stakeholders on the use of skilled care will provide a broad understanding of factors that need to be addressed to increase women’s access to skilled pregnancy care. The objective of this study was therefore, to explore the perspectives of policymakers and health workers, two major stakeholders in the health system, on facilitators and barriers to women’s use of skilled pregnancy care in rural Edo State, Nigeria.

**Methods:**

This paper draws on qualitative data collected in Edo State through key informant interviews with 13 key stakeholders (policy makers and healthcare providers) from a range of institutions. Data was analyzed using an iterative process of inductive and deductive approaches.

**Results:**

Stakeholders identified barriers to pregnant women’s use of skilled pregnancy care and they include; financial constraints, women’s lack of decision-making power, ignorance, poor understanding of health, competitive services offered by traditional birth attendants, previous negative experience with skilled healthcare, shortage of health workforce, and poor financing and governance of the health system. Study participants suggested health insurance schemes, community support for skilled pregnancy care, favourable financial and governance policies, as necessary to facilitate women’s use of skilled pregnancy care.

**Conclusions:**

This study adds to the literature, a rich description of views from policymakers and health providers on the deterrents and enablers to skilled pregnancy care. The views and recommendations of policymakers and health workers have highlighted the importance of multi-level factors in initiatives to improve pregnant women’s health behaviour. Therefore, initiatives seeking to improve pregnant women’s use of skilled pregnancy care should ensure that important factors at each distinct level of the social and physical environment are identified and addressed.

## Background

Global goals are successfully promoting the health agenda with great gains noticeable in regional health-related indicators. The sub-Saharan African (SSA) region achieved a 39% reduction of maternal mortality rates between the years 2000 and 2017 (from 870 to 533 deaths per 100,000 live births) [1]. While substantive, it falls short of the figure needed to achieve the Sustainable Development Goal of 70 maternal deaths per 100,000 live births [1, 2]. Ensuring maternal survival during pregnancy and childbirth is often context specific and dependent on various determinants of health; however, a general consensus is that access to, and the use of skilled pregnancy care ensures the safety of women and their infants [3]. Pregnancy care encompasses care provided to women during pregnancy and at childbirth [4]. Skilled care refers to services by doctors, nurses/midwives and auxiliary nurses/midwives who have been trained to either manage uncomplicated pregnancies, childbirth and post-partum care; or identify, manage or refer complications during any of these periods [5].

Evidence abounds on the relative lack of an enabling environment for skilled pregnancy care in Nigeria. While this has been reported in all parts of the country, there are major urban-rural disparities [6, 7]. In the 5 years preceding Nigeria’s 2018 Demographic Health Survey (DHS), there is evidence that 56% of rural women received antenatal care from skilled providers compared to 84% of urban women. An even lower proportion of rural births (26%) occurred in a health facility compared to 61% of urban births. Furthermore, only 28% of births in rural areas were assisted by a skilled birth attendant compared to 68% in urban areas [8]. With the low use of skilled pregnancy care, it comes as no surprise that Nigerian women are paying a heavy price as seen in the very high numbers of maternal deaths. In 2017, Nigeria recorded 67,000 maternal deaths [1]. Maternal access to and use of skilled pregnancy care is crucial in reducing maternal deaths.

To improve access to, and the use of skilled pregnancy care through primary healthcare (PHC), health interventions such as the Midwives Service Scheme (MSS) and Subsidy Reinvestment and Empowerment Program Maternal and Child Health (SURE-P MCH) were introduced by Nigeria’s federal government in 2010 and 2012 respectively [9, 10]. These interventions focused on increasing human resources for the healthcare sector and upgrading healthcare facilities. They also included advocacy programs by multi-sectoral stakeholders to eliminate healthcare user fees for pregnant women and children and conditional cash transfer schemes aimed at rural women [11, 12]. Even with these strategies, the uptake of skilled care for maternal health remains a challenge thereby pointing to a wider spectrum of factors affecting women’s use of skilled pregnancy care.

Various studies have identified a wide range of factors that determine women’s use of skilled pregnancy care [7, 13–15]. Consistent with the socio-ecological theory of health which posits that individual, community, institutional and policy-related factors influence health seeking behaviour, these studies have attributed the use of skilled pregnancy care to factors such as gender inequality, maternal education, decision-making power, male and community involvement, among others [16]. These factors are mainly associated with individual and community level factors within which pregnant women operate. However, factors that operate at institutional levels, such as health policies are less often explored in relation to women’s use of skilled pregnancy services.

A well-functioning health system is expected to improve access, coverage, quality, and safety of maternal health interventions including skilled attendance at birth. The WHO describes a health system as consisting of all the organizations, institutions, resources and people whose primary purpose is to improve health [5]. A well-functioning health system creates an enabling environment in the provision and uptake of skilled pregnancy care by ensuring that structural elements such as health personnel, medical supplies, financing, and governance are available, functional and optimal [17]. Crucial to the functioning of a health system are the wide range of human actors (stakeholders) such as policy makers, leaders of health professionals and institutions, civil society groups, and healthcare providers, all of who make decisions that shape the delivery and uptake of skilled care. Moore et al. ^18^, contend that advocacy and partnership efforts to improve the health system often focus on healthcare providers with the near exclusion of other stakeholders such as policy makers. However, the ultimate decision making about skilled health delivery approaches and conditions often lie with policy makers and not only healthcare providers [18]. Policy makers and healthcare providers are key managers of the healthcare system, they establish and operationalize the framework within which skilled care is provided and are therefore a reliable source for exploring the individual, community, and institutional landscape of an issue. When key stakeholders are informed and engaged in efforts to improve maternal health, systemic changes are more likely to be achieved and efforts towards improving maternal health more impactful [19]. Therefore engaging policy makers and healthcare providers is necessary to better identify challenges, improve implementation of maternal health interventions, and provide accountability of resources and results [19–21].

Against this backdrop, it can be argued that the perceptions of various influential stakeholders on the use of skilled pregnancy care will provide a wholesome understanding of factors that determine patterns of health behaviours such as pregnant women’s use of skilled healthcare services in rural areas. Qualitative studies across rural Nigeria have examined individual-level and community-level factors associated with the use of skilled pregnancy care [6, 7, 13, 22, 23]. This study extended these previous works by exploring factors that operate at institutional and policy levels that are not commonly explored in relation to women’s use of skilled pregnancy care. The objective of this study was therefore, to explore the perspectives of key stakeholders in the health system on the facilitators and barriers to women’s use of skilled pregnancy care in rural Nigeria.

## Methods

### Study design

A qualitative research design involving key informants at district levels was used. This study forms part of a larger initiative in Edo State by the Women’s Health Action Research Centre and the University of Ottawa, funded under the Innovating for Maternal and Child Health Africa initiative (a partnership of Global Affairs Canada, Canada’s International Development Research Centre and Canadian Institutes of Health Research). As part of the formative phase of the larger study, this study was designed to inform the development of interventions for improving access to, and the use of primary health care services for maternal health in rural Nigeria.

### Research setting

This study was conducted in Esan South East (ESE) and Etsako East (ETE), both of which are local government areas (LGA) of Edo state, one of Nigeria’s thirty-six States. Edo state was chosen because it is one of the lowest performing state in terms of developing and maintaining its PHC system. ETE is in the northern part of Edo State, while ESE is in the southern part. Both LGAs are located in mainly rural parts of the State with each having 10 political wards. ESE comprises of 16,500 residents per ward, while ETE has 14,500 per ward. The principal source of pregnancy care in the two LGAs is primary healthcare. There are 25 PHC centres in ESE and 28 in ETE. Esan South East has one general hospital in the local government’s headquarters (Ubiaja) and Etsako East has two general hospitals; one in the local government’s headquarters (Agenebode) and another in nearby Fugar City. They are used in addition to existing PHCs for referral for maternal health services.

### Participants and recruitment

The study participants consisted of 13 stakeholders from a range of institutions in ESE and ETE LGAs in Edo State, and at the Edo State government level. They included: a senior official within the State Ministry of Health, a senior official within the State Primary Healthcare Development Agency (SPHCDA), Senior officials responsible for PHCs at the LGAs, Senior local government officials, and clinical managers. A purposeful criterion sampling technique was used to select key informants [24]. In this sampling technique, participants are chosen because they meet or exceed a specific criterion (or criteria) related to a phenomenon of interest and therefore possess the knowledge and experience to provided information that is both detailed and generalised [24]. The participants were purposefully recruited by the lead investigators (FO, WI, LN) to represent different backgrounds and professions. The lead investigators contacted each participant by email (or phone) with information about the study and sought their voluntary participation and informed consent. The criteria for participation was that key informants occupy a key position and had experience within the PHC system thus enabling them offer rich insights for addressing the study objectives.

### Data collection and procedures

Interviews were conducted in English by trained investigators. The lead investigators (FO, LN) and members of the technical team, who are experts in qualitative research, provided a three-day training session for the investigators. Aspects of the training included the goals of the research, the art of qualitative data collection, specifically the use of KII guides in qualitative research, the role of the data collectors, research ethics, and data collection using electronic devices such as a voice recorder. Written informed consent was sought and obtained from each study participant prior to starting the interviews. The KII guide was developed by the lead investigators and reviewed for quality assurance. On the last day of training, the trained investigators moderated the pilot of the KII guide in a community with similar characteristics to the study locations.

Data collection using a KII guide took place from July 16 to August 30, 2017. Trained investigators audio recorded the interviews and took reflective field notes to supplement the transcripts. Interviews lasted for 45 mins on average and ended when no further issues arose.

### Research instruments

The KII guide developed for this study (see supplementary file) consisted of open-ended questions and follow-up probes on stakeholders’ perceptions of pregnant women’s use of skilled pregnancy healthcare in rural ESE and ETE communities. Questions explored opinions on comprehensive factors that influence pregnant women’s use of skilled healthcare services. A sample of issues discussed with participants include:

1. The state of maternal health in rural Edo and where pregnant women go to seek care during pregnancy, childbirth and postpartum.
2. How pregnant women’s physical and sociocultural environment influence their use of skilled healthcare in rural areas of Edo.
3. The state of primary health care in rural Edo and challenges and opportunities for effective delivery of healthcare services.
4. Health policies within the PHC framework.

### Ethical considerations

The ethical clearance approval needed for the larger project was obtained from the National Health Research Ethics Committee (NHREC) on April 18, 2017 (reference number NHREC/01/01/2007–18/04/2017). All personal identifiers were removed to ensure confidentiality. Participants provided written informed consent prior to participating in this study.

### Data analysis

The in-depth interviews were audio-taped and transcribed verbatim in the original language which was English. The primary author (OU) and corresponding author (SY) analysed the data and the co-authors validated the data. To ensure accuracy, transcripts were compared with the audio-recordings and field notes. Thematic coding was applied using an iterative process of inductive and deductive approaches. In inductive approaches, themes emerging from the data were fitted into preconceived categories. For this process, Braun and Clark recommend six steps for analysing data; become familiar with the data, generate initial codes, search or themes, review themes, define themes and produce a report [25]. Deductive approaches were drawn from existing literature or theories to build on already studied concepts.

Themes were generated in the following steps: Line-by-line reading generated words or phrases with similar meanings that were linked to the study’s objective and existing literature on pregnancy care in rural Nigeria. These were categorized and noted in the margins of the transcripts. These categories were further grouped into a coding scheme and used to create sub-categories. Subcategories were further merged into larger subcategories with a more general description of the content. Similarities among the larger subcategories were noted and grouped to formulate main categories or themes. Multiple coders (SY, OU) worked independently to analyse the transcript, code the interview data using free codes and develop the various themes. The independent processes were examined for consistency during frequent discussions with the two coders (SY, OU). This was necessary to establish inter-rate reliability and ensure trustworthiness of the study. The co-authors audited the data analysis findings and reached a consensus on emerging themes.

The socio-ecological theory of health behaviour emerged as an organizing framework for presenting the data. This theory asserts that human health behaviour is determined by the interplay of multiple levels of influence across individual, community and the broader institutional and policy realms [16]. Through this theory, this study explored the dynamic interactions of intrapersonal (individual), interpersonal and community, institutional and policy factors that influence the use of skilled pregnancy care. Themes and sub-themes were organised as follows:

I. Individual-level factors: influences of individual characteristics. Sub themes include beliefs, financial status, decision making power, control of financial resources.
II. Interpersonal and community-level factors: influences of formal and informal social systems and networks. Subthemes include cultural, social interactions.
III. Institutional and policy: influences and implications of the current health care system, laws and policies at the local and national levels. Sub-themes examined healthcare workforce, governance and financing, healthcare policies.

### Trustworthiness

The authors adopted various strategies to ensure trustworthiness in this qualitative study following suggestions by Shenton ^27^. KIIs were structured to allow for iterative questioning including the use of probes to elicit detailed data and rephrasing questions to participants when necessary [26]. After data collection, FO and LN conducted member checks to ensure accuracy of the data. The coding process involved two coders (SY, OU) working independently to code the data and collaboratively to generate themes. The principal investigators FO, SY, and LN who have ample experience in reproductive health in sub-Saharan Africa audited the findings and provided feedback. All authors reached a consensus on emerging themes. In writing up the manuscript, the primary author (OU) provided thick descriptions of the phenonmenon of interest. Triangulation is important in promoting confirmability. This study approached triangulation via data sources by interviewing a wide range of informants [26]. In addition, selected quotes were chosen and reported to represent a typical response relative to the theme. To ensure confirmability, the decisions made in the research process including the research objectives and interpretation of findings are described in detail with examples of direct quotations to confirm interpretations [27].

## Results

### Individual-level factors

#### Barriers

Stakeholders (policy makers and healthcare providers) perceived that using skilled pregnancy care was desirable among pregnant women but challenging due to the financial barriers. Financial barriers meant that women were unable to pay for healthcare services even when the costs were not excessive. Participants attributed financial barriers to the high levels of poverty and scarcity of funds among women in rural areas. Poverty, as indicated by the socioecological theory, is the property of an individual and impacts on the ability to afford healthcare. The lack of financial resources, as stakeholders observed, makes the use of traditional birth attendants (TBAs) appealing to women with scarce financial resources. Compared to the average sum of N5000 (~ 13.00USD) required for skilled pregnancy care, TBAs were willing to perform services in exchange for gifts.

*“These services should come free and they do not come free. Even though it is very little, it will take a huge chunk from their pocket because the rural dweller is not an affluent person. Most of them, ignorance does not allow them to also see any need to spend so much on orthodox care. They prefer the cheap roadside carriers.”* (Senior official, PHC, ETE)

Closely tied to the issue of finances is women’s lack of decision-making power and control of financial resources. Participants indicated that men would often override their spouse’s decision-making power and determine their health seeking behaviour if they are covering their spouse’s financial expenses. Participants also observed that women who lacked control of financial resources often feared repercussions of misusing funds from their husbands. Stories were told of domestic violence against women who chose skilled pregnancy care instead of a cheaper alternative. Some men considered funds “misused” if their spouses did not choose a cheaper alternative. Respondents believed that this fear would prompt the women to rely entirely on their husband’s decision on where to seek care.

*“Some of them their husbands abandon them when they come to deliver and refuse to give them food when they get home. I can point about three to four women their husbands abandoned for me when they delivered there [PHC]. I have to make the sacrifice, they left them there [PHC] for me for days, I had to bathe the woman, bathe the baby, feed the woman, feed the baby, their husbands refused to come to discharge them, that the money they will use in feeding them at home is what they want to come and use in paying for them to discharge them, so they abandon them. Some come when they are forced by the community, then when the woman gets home they will starve her, they will refuse to give her food because they have spent the money they are supposed to use in cooking pepper soup for her in the hospital.”* (Clinical manager, ETE)

Participants also commented on the role of women’s belief system in prioritizing skilled pregnancy care. They opined that even when health promotion and awareness strategies are put in place, some women simply believe that native drugs are better. Participants also believed that illiteracy and low levels of education among some of the women were barriers to seeking skilled pregnancy health care. Women who did not understand the importance of skilled care did not think they needed it. Some participants cited similar trends in other parts of Nigeria where levels of education are low. They pinpointed the low use of skilled pregnancy care among pregnant women with low levels of education, and observed that in some cases, the need for skilled care arose only when women experienced complications that could not be handled by traditional birth attendants.

*“There are some that are defiant, I won't go for antenatal, I don’t believe in it. They go to prayer houses, they go to TBA. You know, it is closer, cheaper. A few percentages don't believe in it in the first place.”* (Senior official, PHCs, ESE)

#### Facilitators

Respondents commented on the potential for an appropriate health insurance model to facilitate women’s use of skilled pregnancy care. Nigeria currently has a National Health Insurance Scheme which stakeholders alleged is limited because it only provides coverage for public servants with the government. They claimed that individuals employed in the private and informal sectors are excluded in this model. Study participants acknowledged that out-of-pocket costs are a hinderance to pregnant women’s use of skilled care in rural areas of Edo. They recognised the benefit of a more inclusive insurance model for individuals employed in informal sectors and in rural areas. Some policy makers were privy to ongoing conversations at the state government level on implementing health insurance schemes.

*“The national health insurance scheme has not really embraced the lower or poorer population or rural dwellers. I think it is just for some career staffs of the federal and state governments, so these are just very minute population of Nigeria and so the huge rural population, the huge number of women are not covered by the national health insurance scheme.”* (Senior official, PHCs, ETE)

*“The state government is also looking at that, you know we only have the national health insurance scheme, which is being operated by the national government, by the federal government. The state government is making plans to kick start state health insurance scheme, and under that platform we are going to be having the community health insurance scheme,”* (Senior official, Ministry of Health)

However, some respondents warned of possible challenges to implement insurance schemes, for instance, people could be unwilling to contribute financially to a scheme that could potentially benefit others more than themselves. Stakeholders emphasized the need to provide adequate information to build the trust of women and their communities prior to implementing health insurance schemes.

*“It will not work because if you put that in place where the women will have to contribute, most of them will feel that, they will tell you my husband can pay my bills when I deliver so why should I come to contribute money to be used in paying another person’s bills.”* (Clinical manager, ETE).

*“But there are going to be challenges. We have not come out with such a policy even if we are going to come out with that, there has to be trust, serious advocacy, when you tell people to put in money and there will be counterpart funding, you must be very sure that that thing will take place.”* (Senior official, PHCs, ESE)

To sensitize pregnant women on the importance of skilled care, some stakeholders suggested actively educating women through various media. They believed this would bring about a change in pregnant women’s health seeking behaviour and facilitate their use of skilled healthcare.

### Community-level factors

#### Barriers

All participants identified the competitive practices of TBAs as barriers to women’s use of skilled pregnancy care. TBAs are well established women in the community who offer pregnancy and childbirth services but with no formal training. They are often accessible at all hours, affordable and culturally acceptable to women in their communities. Respondents explained that services rendered by TBAs were more financially affordable than orthodox skilled pregnancy care. TBAs receive payments in kind for services rendered and accept monetary payments in installments. Payment options were not available in skilled health facilities. Respondents saw TBAs as the more attractive choice to some women because they are part of their community, speak the same language and share the same or similar cultures and traditions. Respondents mentioned that TBAs provided native maternal health care to women in their homes or in traditional pregnancy centres. Their services range from social support during pregnancy, to assisting with child births, to performing cultural obligations during the pregnancy period.

Furthermore, participants shared that pregnant women’s use of TBAs was a function of the availability of health facilities in a community. Pregnant women in communities without health facilities were more likely to patronise TBAs. Participants generally believed that TBAs shaped pregnant women’s decisions on the use of skilled pregnancy care.

“*However, many of them still think that for this pregnancy, I will go to the TBAs because that is the person I know. For many reasons, especially when the health centre or facility is far, they look at the cost, they look at their time then they look at the familiarity with the environment. If the facility if far, they will rather go to the TBA.”* (Senior official, PHC, ESE)

*“I think the major challenges will be on the area of people still using TBAs, and that is a function of the availability of the health facilities. Like I mentioned earlier, we don't have health centers in some communities, the people readily available to such women are the traditional birth attendants, I think to go around that problem, the first thing is for us to make deliberate efforts to ensure that virtually all the communities are reached in terms of siting health center.”* (Senior local government official, ETE)

According to participants, previous negative experiences with healthcare can deter women’s use of skilled pregnancy care. Healthcare providers indicated that women were less likely to patronise skilled healthcare if their previous experiences were unpleasant. They were aware of instances where women were mistreated and abused by healthcare workers during their visits. They also reported instances whereby healthcare workers were slow in responding to the needs of pregnant women or ignored them completely. Such delays in receiving care impacted women’s use of skilled healthcare. Healthcare providers argued that women were likely to seek other forms of pregnancy care after negative encounters.

*“That’s what I just told you, the relationship of a person to people matters a lot, if for instance I am a patient and I come to meet you and you insult me, I won’t go there next time.”* (Clinical manager, ETE)

#### Facilitators

Several participants cited a positive relationship between healthcare personnel and the community as crucial to women’s use of skilled pregnancy care. Healthcare providers praised the high use of maternal healthcare services by pregnant women in their communities and attributed it to the very good relationship they have nurtured with the community, particularly community elders. Healthcare providers cited examples whereby they consulted elders in the communities when facing challenges or when delivering a new program to ensure it was culturally appropriate. They believed that this relationship increased patronage of the healthcare centre.

*“For that, the relationship is very good because if we are in the community and we are not able to relate well, nobody will patronize that health center, so the relationship is very good, the community accept us, we accept them, at least we work hand in hand. We are not working in isolation.”* (Clinical manager, ESE)

*The other day when they brought something, the first thing when I left here, I went to the palace to inform His highness [Traditional Ruler] that this is coming to your territory, you encourage your people, you give us maximum support. After that we went again to the ward development community, we now summon chiefs in all the quarters, they came there, we let them know what is happening here, so that it will not look like a taboo to them, they should encourage their people to accept, with that they went out to build the relationship.”* (Clinical manager, ESE)

A common perception among the respondents was that pregnant women whose communities supported skilled pregnancy services and projects were likely to use skilled healthcare. Stakeholders gave an example of a community health project organised by a foreign organisation who came to provide free medical services. The community members took ownership of the project; they were engaged and committed to the overall goal of the project. They rallied around to publicise the free healthcare services, provided town halls as venues for meetings, provided healthcare personnel that collaborated with the foreign doctors and nurses. The stakeholders declared the project a success due to the high uptake of the medical services provided.

### Institutional and policy

#### Barriers

##### Workforce

At the institutional level, participants identified the severe shortage of the health workforce as a key barrier to pregnant women’s use of skilled pregnancy care. Many of the stakeholders bemoaned the severe shortage of skilled health personnel such as doctors to handle health emergencies. Participants reported that the lives of pregnant women and their babies were endangered due to the lack of specialised health professionals such as doctors.

*“PHCs, those that are not equipped, not staffed, have no doctor in charge have recorded more complications and sometimes even death of children and mothers.”* (Senior official, PHC, ETE)

Participants also commented on the lack of a diverse workforce cadre in rural areas. Rural areas were not only facing a lack of highly skilled health personnel such as nurses and doctors, but community health workers and nursing assistants were also scarce. Participants identified the need for doctors, pharmacists, health educators, lab scientists, nurses, health centres, community health workers to provide adequate care to pregnant women.

Furthermore, participants were concerned that the shortage of skilled health workers has meant a heavy workload for currently employed health workers. A respondent reported a case whereby a nurse was running 24-h shifts at the community’s health facility, alone. This, they believed, undermined the provision and delivery of pregnancy care. A senior official with the Ministry of Health provided a sobering example:

*“So right now, there is a doctor covering a whole local government. There are 18 local governments, so we have about 17 doctors, [one] in each local government, you can imagine one doctor covering a local government with 192 wards. So, you can imagine, there is really shortage.”* (Senior official, Ministry of Health)

*“If they come to the health care facilities, sometimes there is nobody there to attend to them. Most times again, labour occurs at night, these PHCs are not manned, they do not have security men, so the poor nurses can’t stay there.”* (Senior official, SPHCDA)

Respondents mentioned that the shortage of health professionals has led to limited hours of operation at some health facilities. Some health facilities would only operate in the mornings but not in the afternoons and evenings due to staff shortage. This posed a barrier to pregnant women seeking skilled care services.

##### State of the infrastructure

The conditions of the health facilities in rural areas were deemed poor by various policy makers and healthcare providers. They said that while structural facilities were available, they were not functional. There were reports of dilapidated health facilities with no hospital beds for patients. There were several reports of the lack of adequate toilet facilities and water supply in some PHC facilities. The state of health facilities resulted in women not receiving adequate care even when they patronised skilled pregnancy services. Participants reported cases whereby women were discharged too early after childbirth and therefore did not receive the appropriate postpartum care. These women requested to leave facilities due to shortage of basic amenities such as hospital beds. A clinical manager commented:

*“A patient will put to bed, no foam to lie down, because of that the husband will come and request for discharge, you know when a patient delivers, you are supposed to observe that patient for, maybe like some hours, but because maybe no good bed, no comfort for that patient, they will request for discharge and maybe they will want to go home.”* (Clinical manager, ESE)

There were also reports of critical shortage of medical equipment in health facilities. Respondents reported the lack of basic requirements such as surgical gloves and cleaning supplies that are necessary during childbirth. Women who patronized these facilities for childbirth bore the expenses of these basic items. Participants reported that in some instances, women were required to provide cleaning supplies, surgical gloves, beds, and mosquito nets prior to childbirth. They perceived this as a deterrent to using skilled pregnancy care. Some mentioned that in other cases, healthcare staff covered the cost of basic medical equipment themselves.

The lack of medical equipment and technologies in most facilities was also identified as a major barrier. This prevented pregnant women from receiving the appropriate care when they arrived at facilities. Respondents argued that the presence of medical equipment in rural health facilities such as oxygen masks will prevent the need to refer women to advanced health centers which will limit their need to travel. They also observed that even when medical equipment were available, they were defective.

*“Well, if we begin from the equipment we need for the very first contact that we have with the pregnant women to check them, they are not there. They are either old or defaced, you cannot even see their markings or those scales on these equipment. You hardly get the accurate reading of them.”* (Senior official, PHCs, ESE)

##### Healthcare financing

Policy makers and healthcare providers were either optimistic or pessimistic on the matter of healthcare financing. Some participants observed that insufficient government spending on health facilities in rural areas created a barrier to the use of facilities. They explained that financing for PHCs is largely decentralised; PHC financing in rural areas falls under the purview of the Local Government. However, the same Local Government had defaulted in paying salaries to healthcare staff for over a year. This has impacted the staff’s ability to provide optimal care to patients. Most respondents agreed that the issue of salary is paramount because salary is a motivation for healthcare staff to carry out their duties, without which, pregnant women will be underserved in these communities.

*“Currently, the PHC is being financed by the local government, they are directly under the authority of the local government, and like I said and you are aware, local government have not been able to pay staff well over a year, so of course, the workers are not happy. Many of them are not going to work, if you go to the PHC, you won’t find a nurse there because she has not been paid. She will tell you that she doesn’t have transport-fare to go to work and you will not blame her. So, salaries have not been paid, basic equipment, drugs are not in supply, everything is at a standstill.”* (Senior official, Ministry of Health)

Some respondents praised their current Local Government for regular salary payments. Even though salaries were not always paid out in full, they believed that it was a better alternative to not getting paid at all. Receiving salaries, albeit incomplete, was a morale booster for healthcare staff. Respondents believed that the Local Government was doing its best amid financial constraints.

*“This LGA does not owe me in terms of salary, which is a positive. If for my salary I am expecting 1 Million and they tell me my salary is going to be 200,000 and am getting it regularly, it is better than not getting it at all. You know, your salary is promised to be 100 Million, and then you are not getting it in the first place. So as far as this local government, they are doing well, and many other local governments. The government has done their best with various fund that have come recently with spread. So that is a major booster. I think it is also a policy that salary, regular salaries are being paid.”* (Senior official, PHCs, ESE)

Another barrier to the use of skilled pregnancy care identified by participants was the fragmented management of the PHC system. They explained that the PHC buildings are the responsibility of the Ministry of Local Government and Community Affairs, while the health programs in the PHCs are administered by the State Ministry of health*.* The staff at PHCs are employed under different Ministries and Departments of both the State and Local Governments. Respondents believed that the challenges of a fragmented system have led to uncoordinated and poor governance which in turn has resulted in gaps in PHC infrastructure, human resources management, and access to basic medical equipment. It was posited by several of the respondents that a combination of these factors influences pregnant women’s use of skilled pregnancy care in rural areas.

*“There is so much fragmentation. Do you understand? There are so many issues, so right now we are trying to get it to be under one management, under one authority. So that there is one body regularizing everything, there will be one body in charge of discipline. So, somebody doesn’t come to work, he is going to be disciplined. Somebody ensures that things are in place, there is security, there is equipment, there is manpower, there is funding. By the time there is a body in charge of all that, less interference from politicians, I think this PHC will actually work.”* (Senior Official, SPHCDA)

### Financing policy

#### Facilitators

Some of the respondents identified beneficial policies that could improve the state of PHCs in rural areas. A policy currently under consideration in Edo State namely, Primary Healthcare Under One Roof, aims to reduce fragmentation in PHC management. Several respondents agree that integrating PHCs under one management will enable facilities run effectively which will in turn improve women’s use of health facilities and reduce maternal death. One respondent explains:

*“By domesticating the policy of primary health care under one roof, we can make our PHCs run effectively. You will be able to improve on your health indices, like the one you are looking at, if you want your PHCs to run effectively, the women will not prefer to go to TBAs they will come to your health facilities and because we have deliveries by skilled attendants, you will also reduce the maternal mortality and the perinatal mortality and the morbidity, that’s the focus of the government and we are working on getting that seriously working, that is if our PHCs are working effectively.”* (Senior official, Ministry of Health).

Furthermore, participants deemed the consolidated policy as financially favorable to PHCs. They commented that with this new policy, PHCs can receive funding from the Federal Government through the Consolidated Revenue Fund (which is the gross federal revenue) for essential drugs, maintaining PHC facilities and equipment, transportation, and for strengthening human resource capacity. Policymakers believed that this would improve the PHC system and maternity services.

*“In the move of all the services for PHC under one roof, there is supposed to be a shared responsibility to take care of that between the local government and the state government, and then also, it will also give them the opportunity to be able to access the consolidated funds from the national, you know, which is one percent of the national budget, that is supposed to fund primary health care across the country.”* (Senior official, Ministry of Health)

## Discussion

This study explored perceptions of key stakeholders on the individual, community and institutional factors affecting the use of skilled pregnancy care in rural Edo State. It sheds light on the barriers and facilitators to skilled pregnancy care from the perspectives of key stakeholders who influence health service delivery. This study compliments other studies that have explicitly studied facilitators and barriers to skilled pregnancy care in rural areas [28–32]. Furthermore, compared to other studies that have mainly investigated individual and community-level factors that influence the use of skilled pregnancy care [6, 7, 13], this study investigated institutional and policy-level factors that influence pregnant women’s use of skilled pregnancy care.

Policymakers and health workers identified some barriers to pregnant women’s use of skilled pregnancy care. First, the respondents perceived that many women were unable to access care due to financial constraints. Accessing skilled pregnancy care at health facilities often entails user fees for consultations, basic equipment related to childbirth and for filling prescriptions. They explained that financial constraints were evident among women who were unemployed and most importantly among women who were living in poverty. The socio-ecological approach identifies an individual’s economic status or access to financial resources as characteristics that could influence an individual’s health behaviour and health outcomes [16]. While these outcomes alone do not determine an individual’s health behaviour, they interact with other factors at different levels to influence health behaviour. Similar findings from Nigeria indicated that poverty was associated with poorer health behaviours among pregnant adolescents and women residing in rural areas [33]. Women and girls living in poverty were less likely to access pregnancy care due to financial constraints.

Furthermore, participants identified women’s lack of decision-making power as a barrier to their use of skilled pregnancy care. This was often mentioned in relation to their lack of access to resources. Key stakeholders perceived that men were the major decision makers for pregnancy care because they provided the financial resources for health care. However, similar studies in Nigeria have linked women’s lack of decision-making power to cultural and social norms whereby the decision of the male spouse takes precedence over the female’s [13, 34]. Furthermore, participants revealed that some women faced domestic violence from their spouses when they used skilled pregnancy care instead of a cheaper alternative. Access to skilled care is influenced by the intersection of access to resources and autonomy in decision making. A review of determinants of women’s use of skilled facilities in low resource settings indicated that gender inequality is a cross cutting determinant of health that operates in conjunction with other determinants [35]. Gender dynamics which manifests in women’s lack of decision making is embedded in women’s access to financial resources and therefore affects her use of skilled pregnancy care. A study in Uganda confirmed that women’s lack of control over financial resources was a barrier to their use of skilled healthcare [36].

Participants identified ignorance, poor understanding of health and lack of awareness as deterrents to using skilled maternity care. This was corroborated by literature [31, 37, 38]. Contrary to studies that indicate improvement in pregnant women’s use of skilled pregnancy care with high awareness and maternal health education [35], the opinions of policy makers and healthcare providers in this study showed that even with health education campaigns in ETE and ESE communities, the use of skilled healthcare remained low. This implies that in some rural areas, there are often multiple factors at play that influence health seeking behaviours.

Furthermore, while generally reasserting the importance of navigating financial difficulties for healthcare, participants were not adequately informed of the different programs under Nigeria’s National Health Insurance Scheme (NHIS). Participants reported that Nigeria’s NHIS does not include coverage of the informal sector, however, the scheme which was operationalised in 2005 with the aim of reducing the burden of rising healthcare costs, operates under three different programs namely; the formal economic health sector, the informal economic sector program and the vulnerable group program [39]. Currently, only 3% of Nigeria’s population are beneficiaries of the scheme with the formal sector accounting for almost all of the total enrolled beneficiaries [40, 41]. Reasons for this has been attributed to inconsistent and poor efforts by the NHIS cooperate body to raise awareness of the prepayment scheme, particularly in a climate rife with poor trust in government. This signifies the need to intensify awareness around health insurance in Nigeria.

Participants in our study believed that rural women’s participation in health insurance schemes will facilitate their use of skilled pregnancy care. Current evidence shows that health insurance programs, particularly community based, can help prevent heavy out-of-pocket health expenditure and improve women’s uptake of maternal health services and deliveries attended by skilled birth attendants [35]. However, uptake of skilled pregnancy care even with insurance coverage has been shown to be a factor of wealth status [42]. Studies conducted in Gabon showed that poor women with insurance coverage were less likely to deliver their babies in a health facility, compared to poor women without health insurance. Rich women with insurance coverage, however, were more likely to deliver their babies in a health facility compared to rich women who did not have health insurance [42]. While this is contrary to stakeholders’ views, it shows the importance of a wholistic approach in exploring interacting factors that influence pregnant women’s uptake of skilled health services. In addition, the unique perspectives of healthcare providers envisage enrollment challenges due to issues of trust and cultural inclinations. Current evidence shows that active efforts to gain the trust of the community will be needed to ensure the success of insurance programs [35, 41].

At the interpersonal and community level, a pregnant women’s formal and informal network, including family, and friendships influences her health seeking behaviour. Similarly, relationships among organisations or institutions such as the relationship between healthcare providers and a woman’s community, all interact to impact her use of skilled pregnancy care.

Participants opined that TBAs were crucial in influencing pregnant women’s health seeking behaviour. They explained that TBAs were deemed affordable, convenient, supportive, and reliable, all qualities that encouraged pregnant women’s patronage and have been confirmed in other studies. In a study on maternal health in 18 low-and middle-income countries [31], reasons for patronising TBA stemmed mainly from women’s perceived quality of care from TBAs. Women emphasised the close bond they felt with TBAs and appreciated the supportive roles TBAs played during pregnancy or childbirth.

Policy makers and healthcare workers perceived that poor provider-patient interactions and delays in receiving care were barriers to women’s use of skilled pregnancy care. They opined that pregnant women would often face mistreatment such as physical or verbal abuse from healthcare worker and that deterred them from using skilled care. On one hand, a systematic review on sub Saharan Africa confirmed that patient mistreatment such as physical abuse affected women’s healthcare seeking behaviour [37]. On the other hand, a study in rural and urban Nigeria found that women and healthcare providers viewed certain mistreatments such as verbal and physical abuse as a necessary and acceptable means to ensure that mothers comply during childbirth [43]. This was to ensure the safety of and positive outcomes for mothers and their babies. In this case, mistreatment did not seem to deter women from using skilled pregnancy care. This study contradicts the perceptions of the stakeholders as reported in this study and may warrant further investigation.

Participants felt that a positive relationship between healthcare providers and communities facilitated women’s use of skilled pregnancy care. The importance of community participation has been acknowledged since the Alma-Ata Declaration of 1978 where families and communities were considered the hub of the health systems [44]. The rationale for community engagement and participation in healthcare emphasises local ownership which is manifested in health programs that respond to the needs of communities and considers how the local context such as cultural practices influence health [44]. A study in Kenya examined the effect of community members partaking in decision making with service providers. The study reported an increase in health indicators such as increase in childbirths in health facilities and improved accountability of service providers to the communities [45].

At the institutional and policy-levels, participants reported severe shortage of healthcare staff in rural areas. Staff were often overworked and not adequately compensated. Health facilities lacked a diverse cadre of specialised health workers which often led to negative patient health outcomes. Participants saw these as reasons why skilled healthcare was often bypassed by pregnant women seeking pregnancy care. These findings were substantiated by studies from across sub-Saharan Africa [37], including a report from Nigeria’s Ministry of Health indicating “shortage and an inequitable distribution of appropriate cadres of health workforce as major barriers to the provision of essential healthcare services” across the country [46]. To address this challenge, the report highlighted the need for task shifting to meet the health needs of the population. Task shifting involves redistribution of tasks, where appropriate, from highly qualified health workers to workers with less training. Participants in this study did not offer this perspective, perhaps because they already expressed concern over the repercussions of unqualified health workers handling obstetric complications. Evidence shows that task shifting maternal health tasks such as basic emergency obstetric care (administration of prenatal drugs, vaginal delivery, neonatal resuscitation) can be done effectively in clinical settings without compromising patient outcomes, however, task shifting is not always sufficient to address complex needs nor is it always sustainable [47]. Applying task shifting would need to be done only in appropriate contexts with health workers receiving adequate training on the specific task they are expected to handle.

The unique perspectives of policy makers highlighted the role of financing and governance policies in women’s use of skilled maternity services. In Nigeria’s health policy [48], primary health care (PHC) falls under the purview of the local government which is arguably the least financially resourced level of government (compared to state and national levels). This has prompted other levels of government to assume some responsibilities for PHCs. The involvement of all three levels of government in PHC has led to diverse management structures with duplicated and poorly defined roles and responsibilities within the different levels of government. The PHC Under One Roof policy aims to eliminate the challenges of fragmented governance by having a state level management agency govern all aspects of PHC [48]. As policy makers explained, Edo state is in the process of establishing an agency that will coordinate PHC activities. They contended that strengthening the leadership and governance structure under the PHC under one roof policy will improve service delivery. A study evaluating the policy in three Nigerian states confirms the benefits of integrated PHC governance [49]. Across three States, Adamawa, Nasarawa and Ondo, the PHC under one roof policy enhanced financial incentives for PHCs to boost provision of services, strengthened community relationships and ownership of facilities, and streamlined roles and responsibilities of PHC governance. Most importantly, the states are experiencing significant milestones such as improved use of key maternal and child health services [49].

Participants noted that the PHC under one roof policy facilitates access to the country’s Consolidated Revenue Fund, however, participants incorrectly noted that PHCs receive 1 % of the national budget when in fact, they receive 45% of the country’s 1% Consolidated Revenue Fund through the Basic Health Care Provision Fund (BHCPF) [50]. The BHCPF was established by the Federal Government in 2014 to facilitate funding to the underfunded PHC sector. The Basic Health Care Provision Fund is predominantly financed from 1% of Nigeria’s total federal revenue before it is shared among the different tiers of government (also known as the Consolidated Revenue Fund). PHCs receive 45% of the Basic Health Care Provision Fund towards ensuring the availability of essential drugs, maintaining infrastructure, providing health transportation and strengthening human resource capacity [50]. The remaining 5% will go towards the provision of medical treatment. However, States and local governments are required to contribute matching funding of 25% of the amount they are receiving through the BHCPF towards PHC projects. Similarly, the remaining 50% of the Consolidated Revenue Fund will be directed towards funding health insurance, particularly for the rural and vulnerable populations [50]. Ensuring women receive timely and quality pregnancy care is challenging in settings where health systems are not adequately funded. Policy makers and healthcare workers suggested that poorly maintained infrastructure, limited medication and essential amenities, unpaid and overworked health workers, higher out-of-pocket cost borne by pregnant women all pointed to a poorly funded health system and called for increased government funding. This is in line with a study that linked government participation in healthcare financing to women’s uptake of skilled birth attendants [51]. The rationale being that government spending will often be evident in better hospital equipment, improved supply of healthcare workforce, and less out-of-pocket spending for patients.

## Conclusion

Findings from this study should be interpreted in light of its limitations. The socio-ecological theory was not employed in its original form due to the availability of data, but the authors grouped together similar themes across similar levels to best capture all its theoretical construct. The study aimed to capture views from a diverse group of stakeholders in Edo State, however, findings may not be generalizable to all of rural Nigeria as ‘stakeholders’ relationships with communities might differ, so will each community’s priorities and experiences with skilled pregnancy healthcare.

The objective of this study was to explore the perspectives of policy makers and healthcare providers on the facilitators and barriers to women’s use of skilled pregnancy care in rural Edo, Nigeria. The socio-ecological theory that guided this study enabled an exploration of multi-level factors that interact to influence women’s use of skilled pregnancy care. This study adds to the literature, a rich description of stakeholder’s views of facilitators to skilled pregnancy care. It also confirms barriers to skilled pregnancy care in rural Nigeria that have been identified in the literature. Findings from this study confirm the significance of factors operating at individual, community and institutional level that interact to influence pregnant women’s use of skilled pregnancy care. The views and recommendations of stakeholders have identified ways to improve provision of skilled pregnancy care. Therefore, initiatives seeking to improve pregnant women’s use of skilled pregnancy care should ensure that important factors at each distinct level of the social and physical environment are identified and addressed. Findings from this study will be useful in modifying existing interventions or designing new interventions that better serve the needs of pregnant women.

## Supplementary Information

**Additional file 1: Supplementary file 1** Key informant interview guide

## Data Availability

The datasets generated and/or analyzed during the current study are not publicly available due analysis being underway for subsequent publications. They are available from the corresponding author on reasonable request.

## Abbreviations

BHCPF: Basic Health Care Provision Fund
DHS: Demographic Health Survey
ESE: Esan South East
ETE: Etsako East
LGA: Local Government Area
KII: Key Informant Interview
NHIS: National Health Insurance Scheme
PHC: Primary Health Care
TBA: Traditional Birth Attendants
SSA: Sub-Saharan African

## References

[1] WHO, UNICEF, UNFPA WBG and the UNPD (2019). Trends in Maternal Mortality : 2000 To 2017. WHO, UNICEF, UNFPA, World Bank Group and the United Nations population division.

[2] WHO (2016). Sdg health and health-related targets. World Heal Stat.

[3] Campbell OM, Graham WJ (2006). Strategies for reducing maternal mortality: getting on with what works. Lancet..

[4] Vanier Institute of the Family (2017). Context: Understanding Maternity Care in Canada [Internet].

[5] World Health Organization. Monitoring the building blocks of health systems: a handbook of indicators and their measurement strategies. World Heal Organozation [Internet] 2010;35(1):1–92. Available from: http://www.annualreviews.org/doi/10.1146/annurev.ecolsys.35.021103.105711.

[6] Solanke BL, Rahman SA (2018). Multilevel analysis of factors associated with assistance during delivery in rural Nigeria: implications for reducing rural-urban inequity in skilled care at delivery. BMC Pregnancy Childbirth.

[7] Ntoimo LFC, Okonofua FE, Igboin B, Ekwo C, Imongan W, Yaya S (2019). Why rural women do not use primary health centres for pregnancy care: evidence from a qualitative study in Nigeria. BMC Pregnancy Childbirth.

[8] National Population Commission (NPC) [Nigeria], ICF (2019). Nigeria Demographic Health Survey 2018. DHS Progr ICF Rockville, Maryland, USA.

[9] World Bank (2014). The evolutions of programmes to increase utilisation of skilled attendance in Nigeria.

[10] Omuta GED, Onokerhoraye AG, Okonofua F, Obanovwe G, Omoraka F, Eregare J, et al. Perspective on Primary Healthcare in Nigeria, Past, Present and Future: Vol. No. 10, CPED Monograph series; 2014.

[11] Okonofua F, Lambo E, Okeibunor J, Agholor K (2011). Advocacy for free maternal and child health care in Nigeria-Results and outcomes. Health Policy (New York) [Internet].

[12] Okoli U, Morris L, Oshin A, Pate MA, Aigbe C, Muhammad A. Conditional cash transfer schemes in Nigeria: potential gains for maternal and child health service uptake in a national pilot programme. BMC Pregnancy Childbirth. 2014;14(408):1-13. Available from: 10.1186/s12884-014-0408-9. 10.1186/s12884-014-0408-9 PMC4273319 25495258

[13] Yaya S, Okonofua F, Ntoimo L, Udenige O, Bishwajit G. Gender inequity as a barrier to women’s access to skilled pregnancy care in rural Nigeria: a qualitative study. Int Health. 2019;11(6):551-60. 10.1093/inthealth/ihz019 31028382

[14] Yaya S, Okonofua F, Ntoimo L, Udenigwe O, Bishwajit G (2019). Men ’ s perception of barriers to women ’ s use and access of skilled pregnancy care in rural Nigeria : a qualitative study.

[15] Okonofua F, Ntoimo L, Ogungbangbe J, Anjorin S, Imongan W, Yaya S (2018). Predictors of women’s utilization of primary health care for skilled pregnancy care in rural Nigeria. BMC Pregnancy Childbirth.

[16] Mcleroy KR, Bibeau D, Steckler A, Glanz K (1988). An ecological perspective on health promotion programs. Health Educ Behav.

[17] Abimbola S, Negin J, Jan S, Martiniuk A (2014). Towards people-centred health systems: A multi-level framework for analysing primary health care governance in low-and middle-income countries. Health Policy Plan.

[18] Mona M K. Rethinking reporductive health communication strategies for the next decade. Geneva: WHO; 1997.

[19] World Health Organization (2014). Multi-Stakeholder Dialogues for Women ’ s and Children ’ s Health : A Guide for Conveners and Facilitators.

[20] Okonofua F (2013). Integrated maternal, newborn and child health (IMNCH) strategy: how has it advanced in Africa?. Afr J Reprod Health [internet].

[21] Ekirapa-Kiracho E, Ghosh U, Brahmachari R, Paina L (2017). Engaging stakeholders: lessons from the use of participatory tools for improving maternal and child care health services. Heal Res Policy Syst.

[22] Okafor IP, Sekoni AO, Ezeiru SS, Ugboaja JO, Inem V (2014). Orthodox versus unorthodox care: a qualitative study on where rural women seek healthcare during pregnancy and childbirth in southwest, Nigeria. Malawi Med J.

[23] Fantaye AW, Okonofua F, Ntoimo L, Yaya S. A qualitative study of community elders’ perceptions about the underutilization of formal maternal care and maternal death in rural Nigeria. Reprod Health. 2019;16(164):1-17. Available from: 10.1186/s12978-019-0831-5. 10.1186/s12978-019-0831-5 PMC6849176 31711527

[24] Patton MQ (1990). Designing qualitative studies. Qualitative evaluation and research methods.

[25] Braun V, Clarke V (2006). Using thematic analysis in psychology. Qual Res Psychol.

[26] Shenton AK (2004). Strategies for ensuring trustworthiness in qualitative research projects. Educ Inf.

[27] Billups F (2014). The quest for rigor in qualitative studies: strategies for institutional researchers. NERA Res.

[28] Uny I, De Kok B, Fustukian S (2019). Weighing the options for delivery care in rural Malawi: community perceptions of a policy promoting exclusive skilled birth attendance and banning traditional birth attendants. Health Policy Plan.

[29] Srivastava J, Joseph A (2019). Why institutional deliveries are low in Balrampur District Uttar Pradesh: a cross-sectional quantitative and qualitative exploration. J Obstet Gynecol India [internet].

[30] Sialubanje C, Massar K, Van Der Pijl MSG, Kirch EM, Hamer DH, Ruiter RAC (2015). Improving access to skilled facility-based delivery services: Women’s beliefs on facilitators and barriers to the utilisation of maternity waiting homes in rural Zambia. Reprod Health [Internet].

[31] Bohren MA, Hunter EC, Munthe-Kaas HM, Souza JP, Vogel JP, Gülmezoglu AM (2014). Facilitators and barriers to facility-based delivery in low- and middle-income countries: a qualitative evidence synthesis. Reprod Health.

[32] Chol C, Hunter C, Debru B, Haile B, Negin J, Cumming RG (2018). Stakeholders’ perspectives on facilitators of and barriers to the utilisation of and access to maternal health services in Eritrea: a qualitative study. BMC Pregnancy Childbirth.

[33] Odo AN, Samuel ES, Nwagu EN, Nnamani PO, Atama CS. Sexual and reproductive health services (SRHS) for adolescents in Enugu state, Nigeria: A mixed methods approach. BMC Health Serv Res. 2018;18(92):1-12. Available from: 10.1186/s12913-017-2779-x. 10.1186/s12913-017-2779-x PMC5806240 29422062

[34] Bamiwuye SO, de Wet N, Adedini SA. Linkages between autonomy, poverty and contraceptive use in two sub-Saharan African countries. Etud Popul Africaine. 2013;27(2):164-73.

[35] Elmusharaf K, Byrne E, O’Donovan D (2015). Strategies to increase demand for maternal health services in resource-limited settings: challenges to be addressed. BMC Public Health.

[36] Morgan R, Tetui M, Muhumuza Kananura R, Ekirapa-Kiracho E, George AS (2017). Gender dynamics affecting maternal health and health care access and use in Uganda. Health Policy Plan [Internet].

[37] Geleto A, Chojenta C, Musa A, Loxton D (2018). Barriers to access and utilization of emergency obstetric care at health facilities in sub-Saharan Africa: a systematic review of literature 11 medical and health sciences 1117 public health and health services. Syst Rev.

[38] Mezmur M, Navaneetham K, Letamo G, Bariagaber H (2017). Individual, household and contextual factors associated with skilled delivery care in Ethiopia: evidence from Ethiopian demographic and health surveys. PLoS One [Internet].

[39] (NHIS) NHIS. National Health Insurance Scheme [Internet]. Programs. 2020; [cited 2020 Aug 6]. Available from: https://www.nhis.gov.ng/our-services/.

[40] Bankole A, Adewole IF, Hussain R, Awolude O, Singh S, Akinyemi JO (2016). The incidence of abortion in Nigeria Akinrinola. Int Perspect Sex Reprod Health.

[41] Adewole DA, Akanbi SA, Osungbade KO, Bello S (2017). Expanding health insurance scheme in the informal sector in Nigeria: awareness as a potential demand-side tool. Pan Afr Med J.

[42] Sanogo NA, Yaya S. Wealth status, health insurance, and maternal health care utilization in Africa: evidence from Gabon. Biomed Res Int. 2020:1-12. Available from: 10.1155/2020/4036830. 10.1155/2020/4036830 PMC7212326 32461984

[43] Bohren MA, Vogel JP, Tunçalp Ö, Fawole B, Titiloye MA, Olutayo AO, et al. “By slapping their laps, the patient will know that you truly care for her”: A qualitative study on social norms and acceptability of the mistreatment of women during childbirth in Abuja, Nigeria. SSM Popul Heal. 2016;2:640-55. 10.1016/j.ssmph.2016.07.003 PMC5356417 28345016

[44] The Global Fund (GF) (2014). Community Systems Strengthening Framework.

[45] Kaseje D, Olayo R, Musita C, Oindo CO, Wafula C, Muga R. Evidence-based dialogue with communities for district health systems’ performance improvement. Glob Public Health. 2010;5(6):595-610. 10.1080/17441690903418969 20162482

[46] Federal Ministry of Health N (2014). Task-Shifting and Task- Sharing Policy for Essential Health Care Services in Nigeria.

[47] Dawson AJ, Buchan J, Duffield C, Homer CSE, Wijewardena K (2014). Task shifting and sharing in maternal and reproductive health in low-income countries: a narrative synthesis of current evidence. Health Policy Plan.

[48] Eboreime EA, Abimbola S, Obi FA, Ebirim O, Olubajo O, Eyles J (2017). Evaluating the sub-national fidelity of national initiatives in decentralized health systems: integrated primary health care governance in Nigeria. BMC Health Serv Res.

[49] Odutolu O, Ihebuzor N, Tilley-Gyado R, Martufi V, Ajuluchukwu M, Olubajo O, et al. Putting institutions at the center of primary health care reforms: experience from implementation in three states in Nigeria. Heal Syst Reform. 2016;2(4):290-301. 10.1080/23288604.2016.1234863 31514721

[50] Uzochukwu B, Onwujekwe E, Mbachu C, Okeke C, Molyneux S, Gilson L (2018). Accountability mechanisms for implementing a health financing option: the case of the basic health care provision fund (BHCPF) in Nigeria Lucy Gilson. Int J Equity Health.

[51] Kruk ME, Galea S, Prescott M, Freedman LP. Health care financing and utilization of maternal health services in developing countries. Health Policy Plan. 2007;22(5):303-10. 10.1093/heapol/czm027 17681975

