# Supplementary material for: Perspectives of policymakers and health providers on barriers and facilitators to skilled pregnancy care: findings from a qualitative study in rural Nigeria
Source: BMC Pregnancy Childbirth. 2021 Jan 6;21:20. doi: 10.1186/s12884-020-03493-8 (PMC7789224; doi:10.1186/s12884-020-03493-8)
Supplement: Supplementary file 1 — Additional file 1: Supplementary file 1 Key informant interview guide [file 12884_2020_3493_MOESM1_ESM.docx]

**Respondents**: A senior official with the State Ministry of Health, a senior official with the State Primary Healthcare Development Agency (SPHCDA), senior officials responsible for PHC at the LGAs, senior LGA officials, and clinical managers in primary healthcare centres.

Thank you very much sir/ma, for your permission to have this interview on record to enable us to transcribe appropriately for our report. Our project is on increasing women’s access to skilled pregnancy care to reduce maternal and perinatal mortality in rural Nigeria specifically.

| **Topic** | **Questions and probes** |
| --- | --- |
| Opening/ Overview of maternal health seeking behaviour | First, we want to review the state of maternal health in rural parts of Edo State.  Where do pregnant women in rural parts of Edo state seek antenatal, childbirth and postnatal care services?  Probe: Why do you think women chose these services? |
| Overview of maternal care services | Do you have information on the number of women who die during pregnancy as well as children from various medical complications?  How does Edo State fare compare to the rest of the country? |
| Skilled pregnancy care in primary health care centres | What are the major issues surrounding pregnant women’s use of primary health care in Edo state?  How do you think these issues can be resolved? |
| Quality of care | Are you satisfied with the quality of care provided in PHCs to pregnant women and children in the most remote parts of Edo state?  Please explain your response. |
| Financing primary health care | How are primary health care centres currently being financed in the state?  What is the average cost of maternal and childbirth services per delivery in rural PHCs? |
| Infrastructural facilities | Can you comment on the state of infrastructural facilities for rural primary health care centres in Edo state?  How do you think the problem can be corrected? (if applicable) |
| Human resources | Regarding human resources available in rural primary health care centers, do you think they have sufficient health workers?  Please explain your response.  How do you think the problem can be corrected? (if applicable) |
| Healthcare policies | Do you know of any existing laws, or policies and programmes run by the state or local government to promote effective primary health care delivery in the state? |
| Closing | Are there any other issues you wish to raise about the readiness of primary health care centres to deliver maternal and childcare services that we have not mentioned so far?  Thank you very much for your time |
